# Informing social media analysis for public health: a cross-sectional survey of professionals

**DOI:** 10.1186/s13690-023-01230-z

**Published:** 2024-01-02

**Authors:** Becky K. White, Elisabeth Wilhelm, Atsuyoshi Ishizumi, Surangani Abeyesekera, Alhassan Pereira, Brian Yau, Aleksandra Kuzmanovic, Tim Nguyen, Sylvie Briand, Tina D. Purnat

**Affiliations:** 1 Department of Epidemic and Pandemic Preparedness and Prevention, Health Emergencies Programme, World Health Organization, Geneva, Switzerland; 2 Visiting Scholar, University of Memphis School of Public Health, Memphis, TN USA; 3 Immunization Demand Team, UNICEF, New York, NY USA; 4 Department of Communications, World Health Organization, Geneva, Switzerland

**Keywords:** Social media, Infodemic, Emergencies, COVID-19, Social listening, Training, Capacity building

## Abstract

**Background:**

During the COVID-19 pandemic, the field of infodemic management has grown in response to urgent global need. Social listening is the first step in managing the infodemic, and many organizations and health systems have implemented processes. Social media analysis tools have traditionally been developed for commercial purposes, rather than public health, and little is known of the experiences and needs of those professionals using them for infodemic management.

**Methods:**

We developed a cross sectional survey and distributed through global infodemic management networks between December 2022 and February 2023. Questions were structured over four sections related to work-practice and user needs and did not collect any personal details from participants. Descriptive analysis was conducted on the study results. Qualitative analysis was used to categorise and understand answers to open-text questions.

**Results:**

There were 417 participants, 162/417 who completed all survey questions, and 255/417 who completed some, all responses are included in analysis. Respondents came from all global regions and a variety of workplaces. Participants had an average of 4.4 years’ experience in the analysis of social media for public health. COVID-19 was the most common health issue people had conducted social media analysis for. Results reveal a range of training, technical capacity, and support needs.

**Conclusions:**

This paper is the first we are aware of to seek and describe the needs of those using social media analysis platforms for public health purposes since the start of the COVID-19 pandemic. There are key areas for future work and research, including addressing the training, capacity building and leadership needs of those working in this space, and the need to facilitate easier access to better platforms for performing social media analysis.

**Supplementary Information:**

The online version contains supplementary material available at 10.1186/s13690-023-01230-z.

**Table:** 

**Text box 1. Contributions to the literature**
• During the COVID-19 pandemic, analysis of social media conversations about health topics became more common to inform health communication and digital engagement.
• Health authorities and their partners have used commercial tools for social media analysis which were repurposed from commercial marketing purposes to public health analysis.
• A first-ever survey was conducted among those who used social media analysis tools to inform public health, identifying gaps in their utility and in skills analysis need to apply them for infodemic insights development in public health.
• The results point to most needed actions to address training, capacity building and leadership needs of analysts working in public health, thus informing workforce development and analytics platform selection.

## Background

A month before the COVID-19 public health emergency of international concern was characterized as a pandemic [1], World Health Organization (WHO) Director General Dr Tedros announced that we were facing an infodemic [2]. The infodemic that has accompanied the pandemic has resulted in an overwhelming amount of information, including misinformation [3]. The infodemic can impact poorly on health outcomes and health systems [4, 5] and there is evidence that those who are most at risk in an emergency, may be most vulnerable to the infodemic as well [6]. While health misinformation is not new in disease outbreaks and emergencies and can be spread both offline and online, infodemics have come to focus with an increasingly digitized society. This has been especially evident during the COVID-19 pandemic, when social and other digital media has allowed for rapid dissemination of an overwhelming amount of information that can reduce effectiveness of pandemic response efforts and interventions [3]. The COVID-19 pandemic has required coordinated global response like no other health emergency in living memory.

As part of the global health response, the field of infodemiology has grown considerably in response to urgent need. Examining this growth a 2021 review examined the number of traditional news articles referencing the infodemic [7]. While searching in the ten years from 2010–2020, the authors identified 61 news stories. Running the same search for just a one-year period, Jan 2020– Jan 2021, resulted in 14,301 published news articles. Since 2020, WHO has formally trained over 1300 infodemic managers from 142 countries [8], with an additional 6500 certified completions of the online OpenWHO infodemic management course to February 2023 [9]. Other organizations are building capacity at global, national and sub-national levels, and infodemic response teams have been established in many settings including Germany [10], Ghana [11], New York City [12], Finland [13], Indonesia [14] and through the Africa Infodemic Response Alliance [15], and the Pan American Health Organization/World Health Organization[16], amongst others. The infodemic has required workforce capacity building and training, at a time that many in the health workforce and emergency response were already managing multiple roles, new tasks, as well as unpreceded personal and professional challenges. While there has been significant inroads made in training and capacity building, from the WHO [17] and other partners, there is a need to better understand current work practices and training and capacity building needs of those working in infodemic management.

Social listening and integrated analysis to generate infodemic insights are the first step in managing the infodemic [18]. Integrated analysis involves bringing together different data sources to get as comprehensive a picture as practical. These could be digital sources (such as social media data, google trends data or website analytics) or non-digital (such as healthcare worker hotlines, focus group data or talk-back radio data). During the COVID-19 pandemic, the practice of social listening grew significantly with regular social listening reports produced by global organizations [19], regions [15, 20] and countries [13, 21]. Understanding the gist, velocity, volume and underlying drivers of narratives circulating in communities can help to inform pre-bunking and debunking initiatives, fill information gaps, and inform infodemic response [19]. In product marketing and brand management, social listening refers to analysis of conversations on social media and public online forums [22] and many commercial social listening platforms exist for this purpose. In public health, social listening is understood as also involving community feedback mechanisms and may also be called digital community engagement [23]. Moreover, analysis of social media narratives and content often includes a wide array of analyses beyond tools used for marketing [24, 25]. For this reason, in this article, we refer to the online social media listening tools, or online social listening systems, as social media analysis tools and define them as any tool that is used to understand how information and narratives on social media spread and is boosted, as well as how people’s information-related behavior in digital spaces is understood and contextualized. There has been little in terms of best practice guidelines for social listening which have also evolved as the practice grew in recently years. A 2023 WHO and UNICEF report outlines the steps an analyst can follow for social listening including data selection and analysis, and integration of offline and online data [25]. WHO has also convened a global panel of experts to develop an ethical framework for social listening and infodemic management [26].

Social media analysis tools have traditionally been established for commercial purposes and brand management, rather than for public health purposes, [27] which has presented challenges to those working on infodemic response. While some challenges of the use of social media analysis platforms have been reported [23], little is known about the current use of these tools and the needs of infodemic managers seeking to utilize them for public health purposes. In seeking to inform future infodemic preparedness, readiness and prevention, understanding the needs and capacity of those working in infodemic management is vital. This research aims to describe the current use and work practice of social media analysis tools and identify gaps and needs of those working in public health.

## Methods

### Study design

This is an observational, descriptive research study, seeking to understand the current use of social media analysis tool use and work-practice, as well as gaps in technical and workforce capacity. A cross-sectional survey was developed seeking the views and input of those working on infodemic response (see Additional 1). The survey content was developed by infodemic management experts from WHO and UNICEF. A small, internal working group informally tested the survey for understandability, reliability and validity and provided feedback on timing. This involved analysis of the questions by multiple team members, to ensure that the questions we were asking were clear, complete and written to elicit the information we wanted from respondents. Team members repeated completion of the survey to ensure they consistently understood the questions and provided similar answers. This was completed by members testing the survey independently, providing written feedback and then via discussion. Questions asked related to work practice and did not collect any personal details from participants.

To encourage responses, none of the questions were required to be completed. All responses received are included in the analysis, along with the number of respondents completing each. The survey was disseminated digitally from the 2nd December 2022 to the 5th February 2023 in the English language. This research project was submitted to the WHO Ethics Review Committee and exempted from review (WHO ERC number CERC.0184).

### Study sample and recruitment

Participants were sought to participate in the study by existing networks of the WHO Infodemic Management team, in the Department of Epidemic and Pandemic Preparedness and Prevention. The survey was distributed widely, and a snowball sampling approach was employed whereby participants were asked to share the survey with others they know who are involved in work of this nature. The aim was not to recruit a representative population sample, but to reach as many people working with social media analysis for public health as possible. We were recruiting internationally, this type of work is rapidly evolving in public health and is done by people from a range of professions, meaning the population is not well defined. We did endeavour to promote to all global regions. Survey links were directly shared via internal WHO and UNICEF networks, through the global WHO infodemic manager community, via the WHO infodemic management newsflash e-list and through other United Nations partners. Participants were eligible to participate if they had used any social media analysis tools since the start of 2020 for a public health purpose. Exclusion criteria included the use of these tools for purposes other than public health. Eligibility criteria was included on the information page of the survey, by entering the survey, respondents were indicating their eligibility.

### Data collection and analysis

The WHO Dataform platform was used for survey development and dissemination. As no personal identifiable data was collected, all data was non-identifiable. Descriptive analysis was conducted on the study results. Qualitative analysis was used to categorise and understand data in the open text questions. Comments deemed irrelevant (for examples those thanking the organisers for their work) were removed.

## Results

### Participant characteristics

There were 417 total responses to the survey, 162 who completed the whole survey, and 255 who partially completed the survey. Responses were received from people working in all global regions and across a variety of workplaces (see Table 1). The highest number of participants were from the African region (29.9%) and university / academia, or national health authorities were the most common employers. Where participants were working at country level, rather than global or regional level, their country of work was requested. A total of 66 different countries were provided by participants, the most common being Nigeria (*n* = 20), USA (*n* = 17) and Ghana (*n* = 15).

**Table 1:** Global region and workplace of respondents

	**n (%)**
**WHO Region that is focus of work (*n* = 278)**	
African region	83 (29.9%)
Region of the Americas	46 (16.5%)
South-East Asia Region	32 (11.5%)
European Region	44 (15.8%)
Eastern Mediterranean Region	12 (4.3%)
Western Pacific Region	17 (6.1%)
Global	44 (15.8%)
**Workplace (*n* = 169)**	
WHO HQ	9 (2.2%)
University / Academia	38 (9.1%)
Media organization	5 (1.2%)
Civil society organization	28 (6.7%)
WHO Regional Office	13 (3.1%)
WHO Country Office	21 (5%)
UNICEF HQ	2 (0.5%)
UNICEF Regional Office	2 (0.5%)
UNICEF Country Office	6 (1.4%)
Other UN / international organization	17 (4.1%)
Health authority (MoH, CDC, Institute of Public Health) – national level	39 (9.4%)
Health authority (MoH, CDC, Institute of Public Health) – subnational level	23 (5.5%)
Other	45 (10.8%)

**Fig. 1:**
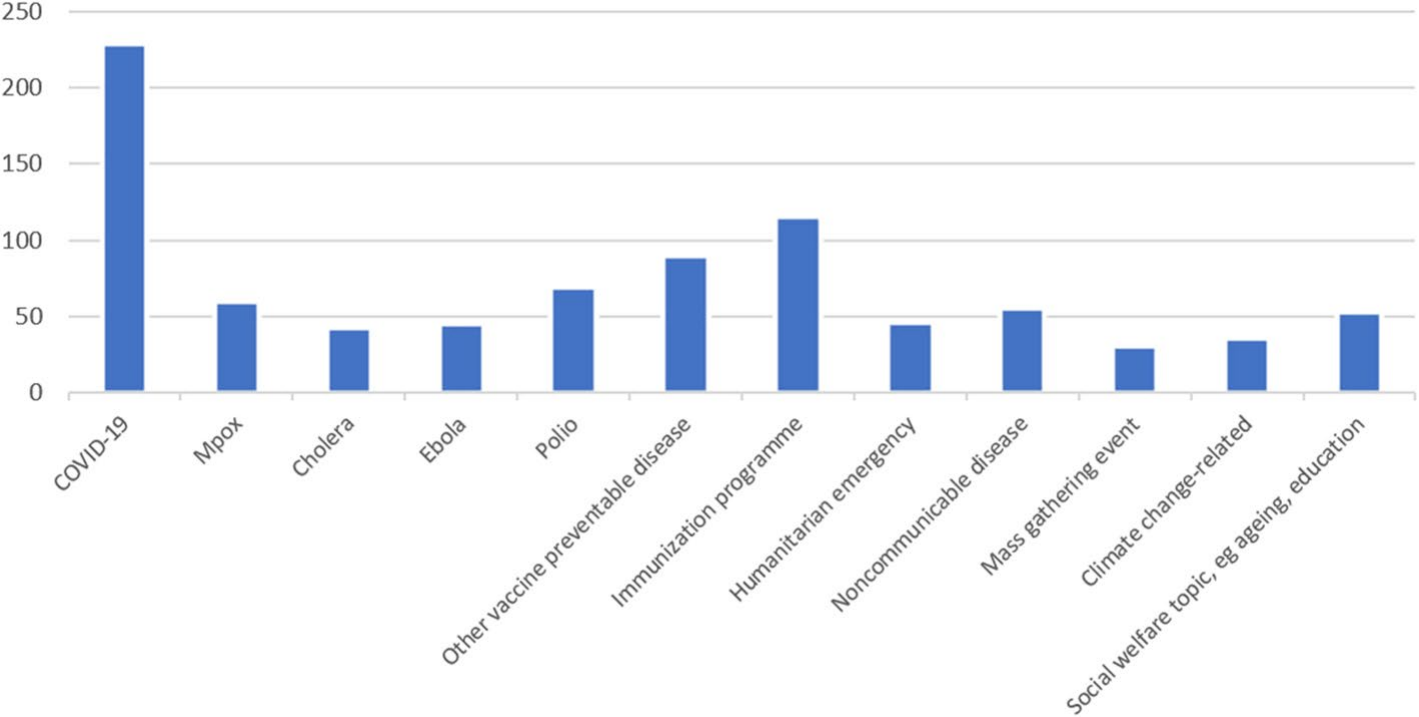
Health topics participants had conducted social media analysis for (*n* = 242)

Respondents had an average of 4.4 years (Std Dev. 4.4) of work experience in the analysis of social media for public health. COVID-19 was by far the most common health topic respondents had conducted social media analysis for. Figure 1 shows the responses (*n* = 242). Outside of the offered options, there were 43 health issues entered in the ‘other’ option, showing the diversity of issues social media analysis is being conducted for. A minority of respondents spent more than 50% of their work time on social media analysis (28/244, 11%), 28% (68/244) reported spending half of their work time on social media analysis while 61% (148/244) said 25% of less of their work time was dedicated to this.

Figure 1 shows the health issues respondents had conducted social media analysis for. Participants could choose multiple options.

Respondents were conducting social media analysis in 56 different languages. English was the most common language currently used (198/324, 61.1%), and the language most wanted to use (67/193, 34.7%). Aside from English, the two languages participants most wanted to cover in social media analysis were French (40/193, 20.7%) and Arabic (31/193, 16.1%). Of the 50 other languages (aside from the 6 UN languages), the most common currently being covered were Portuguese (*n* = 10), Indonesian (*n* = 4), Hindi (*n* = 3) and Swahili (*n* = 4). For those other languages participants most wanted to be available to cover, the most common were Hindi (*n* = 6), Hausa (*n* = 5), German (*n* = 4), Portuguese (*n* = 4), and Swahili (*n* = 4).

### Social media analysis tool use

Respondents asked what specific tasks they currently did, wanted to do, or otherwise. Table 2 shows all responses. For 15/26 individual tasks, over 50% of respondents reported wanting to do the task, but either didn’t know how to, or couldn’t do it with the tools they currently used. The top three tasks’ participants wanted to do but couldn’t with their current tools were: Trace a narrative through social network model and time (45/166, 27.1%); Perform analysis of links to internet web sites or other platforms to detect cross platform switching of content and users (48/162, 29.6%); and Ability to filter between boosted and unboosted/organic content (45/167, 26.9%). While the top three tasks’ participants wanted to do, but didn’t know how to were: Identify bots and bot-generated content (79/164, 48.2%); Automatic scoring of credibility/authority of links in the social media content (81/163, 49.7%); and Identification, filtering and analysis of duplicated content from humans and bots (76/167, 45.5%).

**Table 2:** Tasks respondents currently do, or would like to do on a social media analysis platform (*n* = 173)

Tasks N (%)	Currently do	Want to, not possible with current tools	Want to, don’t know how	Don't want to
Develop own boolean search strings	68 (39.5)	36 (20.9)	53 (30.8)	15 (8.7)
Analyze narratives based on a taxonomy	76 (43.9)	39 (22.5)	50 (28.9)	8 (4.6)
Use filters for gender or user-type	69 (40.1)	41 (23.8)	44 (25.6)	18 (10.5)
Use filters for sentiment or post intent (ie questions / complaints)	78 (46.4)	33 (19.6)	44 (26.2)	13 (7.7)
Filter data to a specific country	116 (65.5)	22 (12.4)	34 (19.2)	5 (2.8)
Filter data to a specific language	104 (59.1)	27 15.3)	34 (19.3)	11 (6.3)
Filter data for specific platform	103(59.9)	24 (14)	34 (19.8)	11 6.4)
Group posts to themes	86 (50)	38 (22.1)	39 (22.7)	9 (5.2)
Export data	105 (61.4)	25 (14.6)	32 (18.7)	9 (5.3)
Annotate narrative themes and content for easier thematic analysis	68 (41.5)	39 (23.8)	49 (29.9)	8 (4.9)
Compare and integrate with other data sources	70 (41.5)	41 (24.3)	48 (28.4)	10 (5.9)
Examine narrative themes and labels by changes in velocity as well as volume	52 (31.7)	38 (23.2)	59 (36)	15 (9.1)
Compare data over time	120 (68.2)	26 (14.8)	27 (15.3)	3 (1.7)
Identify common misinformation narratives	115 9 (67.3)	24 (14)	28 (16.4)	4 (2.3)
Do social network analysis	97 (56.1)	35 (20.2)	38 (22)	3 (1.7)
Trace a narrative through social network model and time	55 (33.1)	45 (27.1)	55 (33.1)	11 (6.6)
Annotate a narrative shift or mutation over time and social connections	49 (29.2)	38 (22.6)	63 (37.5)	18 (10.7)
Identify bots and bot-generated content	31 (18.9)	40 (24.4)	79 (48.2)	14 (8.5)
Import list of trusted users or sources for automated annotation/whitelisting	32 (20)	41 (25.6)	69 (43.1)	18 (11.3)
Import list of dubious users or pages for flagging in the analysis as untrustworthy	33 (20.4)	42 (25.9)	68 (42)	19 (11.7)
Perform analysis of links to internet web sites or other platforms to detect cross platform switching of content and users	35 (21.6)	48 (29.6)	64 (39.5)	15 (9.3)
Automatic scoring of credibility/authority of links in the social media content	27 (16.6)	41 (25.2)	81 (49.7)	14 (8.6)
Identification, filtering and analysis of duplicated content from humans and bots	27 (16.6)	43 (25.7)	76 (45.5)	21 (12.6)
Ability to filter between boosted and unboosted/organic content	38 (22.8)	45 (26.9)	65 (38.9)	19 (11.4)
Compare text based to image or video-based content	54 (31.8)	41 (24.1)	57 (33.5)	18 (10.6)
Compare data between countries or communities	77 (44.8)	37 (21.5)	43 (25)	15 (8.7)

Participants were asked their agreement with a range of confidence and capacity statements (see Table 3). This was presented as a 5-point Likert scale, strongly agree and agree responses, and strongly disagree and disagree responses have been combined. Less than 40% agreed that that social media analysis platform they were using was fully meeting their needs, and only about a third (35.7%, *n* = 65) agreed that the resource allocation for social media analysis was sufficient. A question was posed about resourcing being sufficient to achieve objectives. Organizations would have different objectives, but the majority indicated that these were to understand and respond to the concerns, questions and narratives of the community (see next section).

**Table 3:** Respondent agreement with confidence and capacity statements (*n* = 186)

N (%)	Agree	Neither	Disagree
I feel confident using social media analysis tools to their full capacity	107 (57.5)	37 (19.9)	42 (22.6)
I feel confident integrating social media data with other data including from offline sources	106 (57.9)	39 (21.3)	38 (20.8)
The social media analysis platform we use fully meets our needs	72 (39.1)	50 (27.2)	62 (33.7)
The time spent on social media analysis is sufficient to achieve objectives	80 (44)	39 (21.4)	63 (34.6)
The resource allocation on social media analysis is sufficient to achieve objectives	65 (35.7)	46 (25.3)	71 (39)
Combining social media data with offline data and other sources is important	161 (87.5)	15 (8.2)	8 (4.3)

### Reporting and recommendations based on social media analysis

Most participants indicated they did combine the social media data with other data sources when analysing (103/176, 58%). A number of different sources were used including epidemiological data (*n* = 65), qualitative data from key informant interviews (*n* = 53), community forum data (*n* = 50), and KAP (knowledge, attitudes and practices) survey data (*n* = 50). A total of 54/176 respondents (31%) reported not integrating with other data sources. The most prominent reason was a lack of human resources to source and integrate work, followed by not having enough time, or not knowing how to. Open text responses shared some of the difficulties experienced.

### Aims and purposes of data sources differ too much

*We want first to fully understand all the parameters related to social media analysis in Twitter, then continue to other social media apps and then perhaps engage with other internet sources.*

Users were asked the reason for producing their social media report and what happened with the results. The most common reason was ‘To understand the current questions, concerns and information voids in different online communities and produce rapid content to respond to them’ (115/170), followed by ‘planning communications and campaigns’ (91/170) and ‘to conduct research’ (71/170). Other responses given (*n* = 12) included for briefing leadership and country offices, tracking misinformation, and identifying rumours.

Participants were asked if they formulated recommendations based on their social media analysis, 71% (122/171) indicated they did so, with 19% (33/171) indicating they did not, and 10% (16/171) who did not know. For those who didn’t reasons were a lack of time (33%, 11/33), not knowing how to (21%, 7/33), and it not being a requirement (45%, 15/33).

### Strengthening social media analysis for public health

The final questions asked how social media analysis could be strengthened for those working in public health. The top 5 criteria identified by respondents when choosing a tool for public health purposes were:

1. Cheap or free to use
2. Familiarity of our team with the tool
3. Easiest to procure for our team
4. All colleagues have access to same platform
5. Broad inclusion of many digital sources

**Fig. 2:**
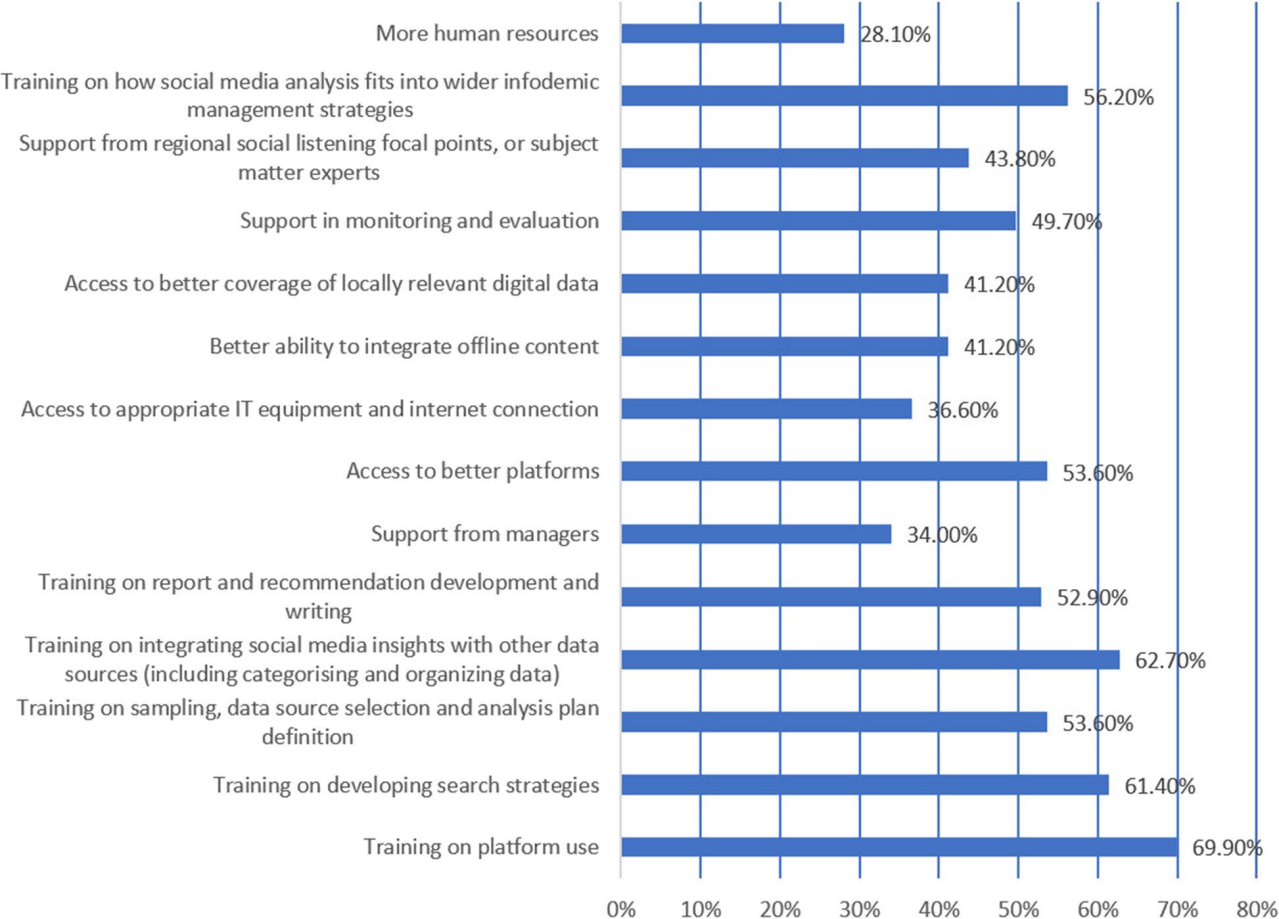
Support or training needs identified as needed by respondents (*n* = 153)

Respondents were asked about their support and training needs. Figure 2 shows that over 50% of respondents indicated the need for additional training and support for half of the options given. The highest training needs were technical capacity building such as platform training (107/153, 70%), integrating with other data sources (96/153, 62.7%) and developing search strategies (94/153, 61%).

Figure 2 shows the training and support needs of participants. Participants could choose multiple options, and results are shown as percentages of respondents choosing each option.

Other options given referred to increased collaboration and peer-support mechanisms, and increased capacity, including needing more time, more logistical support and more funding.

*Coordination with HQ/other regional offices in terms of listening tools/methodology/new listening sources, taxonomy*

*Train consultants and offer a toolkit for demos and prototypes*

The final question was an open-text question asking respondents for final thoughts. After irrelevant comments had been removed, there were 40 written responses. Table 4 shows the key themes identified in a qualitative analysis of the comments along with an example, while all responses received are included in Additional 2.

**Table 4:** Responses to open-text question asking final thoughts on social media monitoring or integrated analysis

Theme	Description	Example
Increasing resourcing and collaboration	A need for collaboration, and coordination at global at regional levels	*Currently, there isn't a data intelligence department or team or capabilities for the technical units. Social media is just one piece of the puzzle, we would like to know what is happening in terms of information and how this affects our department and activities and strategize with research backing our decisions* *To excel in this area, we need to have trained dedicated resources at country offices to work on this area themselves and to work with the national counterparts to strengthen their capacities as well*
Recognising value	Several comments related to managerial barriers and the need to build capacity of those managing infodemic teams to recognise the value	*Managers tend to expect way more results for way less money. Social media analysis is a multidisciplinary venture: data, linguistics, psychology, media-expertise, all is needed to optimally get the potential. This makes it a challenge to acquire and maintain the teams* *I am needing support to help demonstrate value for local public health units because the comms teams are resisting. National associations have an AMAZING social media presence with comments posted—but not being analyzed or integrated to my knowledge*
Advocacy and strategic leadership	Comments in this theme addressed advocacy and leadership needed to advance infodemic management. This included advocacy with platforms	*Sometimes we have the management support and the colleagues willingness to do the analysis or provide the data, and also there are existing platforms that can provide most of our requirements, however the legal process is not as fast as we need or teams can not use or move forward with a platform for legal processes* *We need help to PRESSURE these social media platforms, to open up access to RESEARCHERS—so in turn we can better help make things better in their platform* *Most managers in surveillance programs or national associations that utilize social media are not going past the "counting clicks" part. They aren't using digital data for analysis and recommendation sharing. I look forward to have this advanced in 2023 in collaboration with local, regional and national orgs.:)*
Digital and technical barriers	Some respondents discussed wanting better access to data and to better platforms. Others discussed challenges with internet and VPN access, as well as a lack of data for certain priority groups	*It's still very hard to obtain USABLE data from various social media platforms—TikTok, Facebook, Instagram. For example, we tried to create a crawler for Instagram, to be able to better monitor the infodemic situation in that platform. Instead, we were hit by various very harsh anti-bot measures. We should not need to create the crawler at the first place, but since these platforms are so bent on closing off their places, we had to* *It's very difficult to get data based on rase or ethnicity. My agency targets the Black American audience, which has lower vaccination rates that other communities. We can't filter users by race/ethnicity, and there are very few community reports about vaccination rates that do this. So, a lot of it involves making guesses about race, or using other identifiers to assume their race. I wish there was a way to get more data based on race*
Teaching and evidence	These comments included support for those teaching university level students, as well as calls for computer literacy and social inoculation	*I would like to know any relevant publications which use social media monitoring tools, and how to develop materials for teaching social media monitoring in basic level to equip health cadres with ability to track mis/disinformation* *I teach MPH public health policy, and the importance of the use of good data to justify the policy action. So I am primarily instructing my students on access to use these resources. This educational route was not really mentioned in your survey*
Technical training needs	Respondents detailed technical training needs for themselves and the sector, and called for training to be offered in a range of modalities	*It has been hard to find a way to integrate what the brand needs versus what the technical units need* *Better to work on the following: These are the key social media metrics that should not miss from your social media analysis report: post ranks across all channels. number of posts. post engagement and engagement rate. all campaigns. total campaign engagement. number of posts per campaign. posts volumes by campaign (numerically and percentage wise)* *I would like more training through MOOC*

## Discussion

This paper has outlined the results from a global survey of those working using social media analysis for public health. It included respondents from all global regions and from a variety of workplaces. These results reveal gaps in training and a need to increase capacity building and leadership for those working in this space. They also highlight gaps in technical and public health capability of social media analysis tools. There is a need for easier access to better platforms with technical capacity developed in consultation with, or with the needs of public health professionals at the centre.

The respondents of our survey were those currently working in social media analysis for public health, with an average of 4.4 years’ experience, and across 55 different health issues. These experienced professionals identified a wide range of training and capacity building needs, which suggests the needs of others new to working in the field, or working adjacently, may be much greater. Over 50% of respondents indicated the need for additional training and support for half of the listed options. These ranged from technical need, such as training on developing search strategies and on platform use, to more systemic needs such as how social media analysis fits into the wider infodemic framework, integrated analysis, and monitoring and evaluation support. For 20/26 different listed technical tasks (Table 2), more than one fifth of respondents indicated they wanted to but didn’t know how to do the specific task. Respondents were seeking support with advocating for data and platform availability, for support demonstrating value to managers and other partners, and for global coordination and support. Only 57.5% of respondents felt confident using social media analysis tools to their full capacity, and training on platform use was the top training need identified by 69.9% of respondents.

These calls for increased capacity mirror those reported during a consultation on a competency framework for infodemics which called for greater institutional capacity and ongoing education [18]. While the majority of respondents in our survey agreed that combining social media data with offline data is important (87.5%, 161/184), there are clear gaps in capacity with ‘training on integrating social media insights with other data sources’ being the second highest training need identified by 62.7% of respondents. This integration is a best practice approach advocated by organizations to ensure broader community representation of data used for decision-making [19, 21]*.* More broadly, there have been calls for increased integrated social listening capacity in the health promotion workforce [28], and specific guidance on data sources, integrated analysis and insights reporting has been developed for certain settings, including at a national public health institute level in Germany [10]. A recently released WHO and UNICEF manual outlining steps to generate an infodemic insights report using a systematic approach to integrated analysis of infodemic insights from a variety of data sources will provide practical guidance for those working in the field [25].

The suitability of current platforms to meet the needs of those conducting social media analysis for public health was a key theme. Only 39.1% of respondents agreed that the social media analysis tool they used fully met their needs. Participants demonstrated a desire for a wider coverage of languages. Respondents were conducting their own social media analysis in 56 languages, yet many platforms have a very limited language coverage. Participants reported limitations in data sources and diversity, as well as technical agility. There was a total of 19/26 different tasks (Table 2) that more than one fifth of respondents wanted to do but were not possible with their current tools. Cost was clearly an important factor with respondents listing it as their top criteria in choosing a social media analysis platform. These results show participants want more access to tools that include a variety of digital sources, wider technical functions, and the ability to integrate offline content. Involving public health professionals in the development, or functional review of these tools would be beneficial. As the social media landscape is in flux, with prominent platforms experiencing significant change and the introduction of new channels, tools will need to continually evolve to remain relevant and useful.

These challenges for public health analysts have been reported more widely. A recent study into social listening barriers and recommendations from the COVID-19 response found the representation of social media data to be a key barrier, and the commercial use design a challenge as algorithms, content and features were geared more towards commercial needs, than public health needs [23]. A number of organizations have detailed their approach to social listening and infodemic insights. In Eastern and Southern Africa, UNICEF and partners describe an integrated approach combining offline and online listening to aid the pandemic response, note the challenges of commercially available tools, and call for greater flexibility in integrating with different data sources and the consideration of the development of monitoring platforms [20]. The United States Centers for Disease Control and Prevention describe their process of producing the State of Vaccine Confidence social listening and infodemic insights report and describe the personnel and time required to produce these reports as a limitation [21]. Other research has called for the insistence of partnerships with social media companies and government agencies to further public health [29]. This included ensuring access to data, coordination on policy initiatives and moderation and notification pathways.

It is clear from the breadth of health issues respondents were covering that social media analysis and their integration into infodemic insights are useful outside of emergency response. There is evidence emerging of impact of the infodemic on non-communicable diseases [30] climate change [31] vaccine acceptance [32] and mental health [33], among many other health areas, even being suggested as a “major determinant of health” [34] through its interaction with health literacy [35]. To respond to global issues, we need to look at interconnected solutions and training, with localised options and relevance. Most research about social listening has been conducted in high income countries [4] and this paper adds a more global view of needs. The call for better access to, and ability to integrate local data was clear. The need for coordination was exemplified by one comment from a survey participant who described their attempt to create a crawler for Instagram to better enable monitoring of infodemic situations. Having local teams attempting to create individual solutions is not an effective use of time or capacity. There is a need for more transparent access to data and social media platforms, advocacy to enable this, global collaboration on solutions, and analysis platforms developed in collaboration with those working in public health.

This research has revealed important insights on the needs of those working in infodemic management for public health. To our knowledge, this is the first global survey directly targeting those working on social media analysis for public health during the COVID-19 pandemic. There is more research needed on how analysis of narratives on social media platforms and more broadly in information ecosystem(s) can be conducted and scaled up, how locally relevant and offline sources can be integrated, how public health workers can be supported to do this and how better access to data and better analytical platforms can be facilitated. This paper has described the challenges specifically in the analysis of data form social media platforms. Working directly with those using social media analysis for public health to further define and scope the approaches and tools, including the technical needs of analytical platforms, as well as make specific recommendations for tools and practice will be an important next step.

But broader challenges in metrics and measurement of the circulating concerns, questions, information voids, and narratives, are also required in addition to focusing on social media analytics alone. For example, WHO has piloted a platform for responsible and improved analysis of narratives, concerns and questions expressed by people in digital spaces, while at the same time also explored the opportunities from using digital ethnography and qualitative approaches to understand concerns and narratives circulating in communities. Moreover, WHO and UNICEF have developed a manual and trainings on how to triangulate social media analysis with other health data sources to improve the usability and relevance of infodemic insights. As in any public health analysis, triangulation of intelligence from different data sources strengthens the evidence and recommendations formed based on the analysis. Better social media analytics, with better triangulation of these insights with other health data sources will increase the quality of social listening and infodemic insights generation for public health.

### Limitations

This survey sought the views of those working in social media analysis since 2020. All of the responses given are based on the self-report of participants and were not verified. One example is that over 50% of respondents reporting conducing social network analysis. Further work would need to be undertaken to understand the way this is being conducted. The training and other results described here reflect the views of those respondents, however they will not be representative of those conducting social listening via other means, or prior to 2020. While we have demonstrated broad geographical representation in our participants, the Western Pacific and Eastern Mediterranean Regions were under-represented, this reduced our ability to be able to stratify responses and look between groups. Our recruitment approach of working through WHO networks and then widening out to others mean that there will be people we have not reached, thus these results cannot be generalised. In addition, the survey was only conducted in English, further limiting reach.

## Conclusions

This paper is the first we are aware of to seek and describe the needs of those using social media analysis platforms for public health purposes since the start of the COVID-19 pandemic. There were some key areas for future work and research, including addressing the training, capacity building and leadership needs of those working in this space, and the need to facilitate easier access to better platforms for performing social media analysis. Our findings are in-line with recommendations from other studies but work in this area remains limited. As health systems move to integrating pandemic preparedness planning and capacities into health system capacity planning and emergency plans for future infodemic preparedness, readiness and prevention, understanding the needs and capacity of those working in infodemic management is vital.

### Supplementary Information

**Additional file 1.** Survey questions.

**Additional file 2.** Full list of responses to open-text question asking final thoughts mapped to themes.

## Data Availability

The datasets generated and/or analysed during the current study are not publicly available but are available from the corresponding author on reasonable request.
