# Supplementary material for: Informing social media analysis for public health: a cross-sectional survey of professionals
Source: Arch Public Health. 2024 Jan 2;82:1. doi: 10.1186/s13690-023-01230-z (PMC10759433; doi:10.1186/s13690-023-01230-z)
Supplement: Supplementary file 1 — Additional file 1. Survey questions. [file 13690_2023_1230_MOESM1_ESM.docx]

**Additional file 1. Survey questions**

**Workplace and work practice (Section 1/4)**

The following questions ask about your work and your work practice related to social media analysis. We do not ask any personally identifiable questions.

1. **Which WHO region is the focus of your work? (If working for another organization, please approximate the coverage of the regions)** Please choose **all** that apply:

- African Region
- Region of the Americas
- South-East Asia Region
- European Region
- Eastern Mediterranean Region
- Western Pacific Region
- Global

1. **What country is the focus of your work? (Please leave blank if you work at regional or global level)** Please choose only one of the following: (country list provided)

1. **What is the primary working language in the region / country you work in?** Please choose **all** that apply:

- Arabic
- Chinese
- English
- French
- Russian
- Spanish
- Other: ____________________

1. **Where do you work? Please choose your main workplace or the one that suits you best.** Please choose **only one** of the following:

- WHO HQ
- WHO Regional office
- WHO Country office
- UNICEF HQ
- UNICEF Regional Office
- UNICEF Country Office
- Other UN / international organization
- Health authority (MoH, CDC, Institute of Public Health) – national level
- Health authority (MoH, CDC, Institute of Public Health) – subnational level Media organization
- Civil society organization
- University / Acedemia
- Other ________________

1. **How long have you been involved in the analysis of social media for public health? (Please tell us number of years)**

Please write your answer here: _____________

1. **For which topics have you conducted social media analysis for a health context? Please choose all that apply:**

- COVID-19
- Mpox
- Cholera
- Ebola
- Polio
- Other vaccine preventable disease
- Immunization programme
- Humanitarian emergency
- Noncommunicable disease
- Mass gathering event
- Climate change-related
- Social welfare topic, eg ageing, education
- Other: _________________

1. **Which of the following best describes your work with social media analysis tools? Please choose all that apply:**

- I conduct my own social media analysis for health action using various tools and
- data sources
- I work in a team where we conduct social media analysis for health action together
- I supervise others conducting social media analysis for health action
- I am a researcher
- I work with an external provider who conducts social media analysis on our behalf
- Other: ____________________________

1. **What does your social media analysis work-practice look like? Please choose all that apply:**

- I browse the dashboard for latest posts on a topic
- I analyze thematic narratives on a topic
- I investigate a narrative across online communities, and its evolution over time
- I produce a summary of social media engagement on a topic, and general themes of conversation
- I analyze for acute questions, concerns, information voids, circulating narratives and mis/disinformation
- I review a report provided to me by an external provider
- None of the above

1. **Please estimate what percentage of your time each week is spent on social media analysis work. Please choose only one of the following:**

- 100%
- 75%
- 50%
- 25%
- Less than 25%

1. **Please tell us what language/s your social media analysis currently covers. Please choose all that apply:**

- Arabic
- Chinese
- English
- French
- Russian
- Spanish
- Other: _________________

1. **Please tell us which additional languages you would like your social media analysis to cover. Please choose all that apply:**

- Arabic
- Chinese
- English
- French
- Russian
- Spanish
- Other: _________________

**Social media analysis tools (Section 2/4)**
 The next few questions ask about social media analysis tools and how they are being used.

1. **What social media monitoring tools do you or your team currently use or have used recently? Please choose all that apply:**

- Tweetdeck
- Talkwalker (Free tools)
- Talkwalker (Paid access)
- CrowdTangle (Plug-in)
- CrowdTangle (Full access)
- WHO Early AI Supported Response with Social Listening (EARS) (Public portal)
- WHO EARS (Backend portal)
- Spike
- GoogleTrends
- UNICEF Vaccine Demand Observatory
- Meltwater
- Hootsuite
- Sprout
- Other: _________________

1. **What tasks do you currently do, or would you like to be able to do on a social media analysis platform? Please choose the appropriate response for each item:**

|  | **Currently do** | **Want to, not possible with current tools** | **Want to, don’t know how** | **Don't want to** |
| --- | --- | --- | --- | --- |
| Develop own boolean search strings |  |  |  |  |
| Analyze narratives based on a taxonomy |  |  |  |  |
| Use filters for gender or user-type |  |  |  |  |
| Use filters for sentiment or post intent (ie questions / complaints) |  |  |  |  |
| Filter data to a specific country |  |  |  |  |
| Filter data to a specific language |  |  |  |  |
| Filter data for specific platform |  |  |  |  |
| Group posts to themes |  |  |  |  |
| Export data |  |  |  |  |
| Annotate narrative themes and content for easier thematic analysis |  |  |  |  |
| Compare and integrate with other data sources |  |  |  |  |
| Examine narrative themes and labels by changes in velocity as well as volume |  |  |  |  |
| Compare data over time |  |  |  |  |
| Identify common misinformation narratives |  |  |  |  |
| Do social network analysis |  |  |  |  |
| Trace a narrative through social network model and time |  |  |  |  |
| Annotate a narrative shift or mutation over time and social connections |  |  |  |  |
| Identify bots and bot-generated content |  |  |  |  |
| Import list of trusted users or sources for automated annotation/whitelisting |  |  |  |  |
| Import list of dubious users or pages for flagging in the analysis as untrustworthy |  |  |  |  |
| Perform analysis of links to internet web sites or other platforms to detect cross platform switching of content and users |  |  |  |  |
| Automatic scoring of credibility/authority of links in the social media content |  |  |  |  |
| Identification, filtering and analysis of duplicated content from humans and bots |  |  |  |  |
| Ability to filter between boosted and unboosted/organic content |  |  |  |  |
| Compare text based to image or video-based content |  |  |  |  |
| Compare data between countries or communities |  |  |  |  |

1. **How much do you agree with the following statements? Please choose the appropriate response for each item:**

|  | **Strongly agree** | **Agree** | **Neither agree or disagree** | **Disagree** | **Strongly disagree** |
| --- | --- | --- | --- | --- | --- |
| I feel confident using social media analysis tools to their full capacity |  |  |  |  |  |
| I feel confident integrating social media data with other data including from offline sources |  |  |  |  |  |
| The social media analysis platform we use fully meets our needs |  |  |  |  |  |
| The time spent on social media analysis is sufficient to achieve objectives |  |  |  |  |  |
| The resource allocation on social media analysis is sufficient to achieve objectives |  |  |  |  |  |
| Combining social media data with offline data and other sources is important |  |  |  |  |  |

**Reporting and recommendations based on social media analysis (Section 3/4)** The next few questions ask about reporting and recommendations based on social media analysis.

1. **When analyzing the data, do you compare insights with data sources other than from social media (for example KAP survey data, community feedback, hotlines, immunization registry data, clinical encounter data, feedback from health care workers etc)? Please choose only one of the following:**

- Yes
- No
- I don't know

1. ***(if yes to 15)* Please tell us what other data sources you include. Please choose all that apply.**

- KAP survey data
- Community forum data
- Community hotline data
- Epidemiological data
- Health service demand data
- Health records
- Data from health worker tip-lines, informants and networks
- Mobility data
- Consumer insights
- Qualitative data from key informant interviews
- Radio talkback data
- Qualitative data from focus groups with HCW, communities of focus, etc
- Other: ___________________________

1. ***(If no to 15)* Please tell us why you don’t compare and cross-analyze across different data sources. Please choose all that apply:**

- Don’t have time
- Social media data is comprehensive enough
- There are no other sources available
- Lack of human resources to source and integrate work
- Other data is low quality and not reliable
- Other data is not timely enough
- We don’t know how to integrate effectively
- We don't know where to begin looking for additional data sources
- Other: ___________________________

1. **Which of the following tasks do you do to complete your social media report? Please choose all that apply:**

- Set up search strings on a social media analysis platform
- Generate automatic reports on a social media analysis platform
- Distribute automatic reports from social media analysis platform with colleagues, without further analysis
- Export automatic reports from a social media analysis platform
- Further work with the exported data for linguistic, trend and thematic analysis Compare reports from different social media analysis platforms or tools Compare reports with other data sources
- Other: ______________________________________________

1. **Once you have set up your work in a social media analysis tool, do you do any of the following maintenance tasks? Please choose all that apply:**

- Update search strings to include new relevant keywords or remove outdated ones
- Detect narrative drift from previous reports to identify relevant new keywords or platforms
- Set up new searches
- Identify additional online platforms for inclusion for monitoring and analysis We do not do any maintenance tasks
- Other: ______________________________________________

1. **For what purpose do you use the results of your analysis of social media? Please choose all that apply:**

- To plan communications products and campaigns as part of an editorial calendar
- To investigate an online influencer and their following
- To support reputation or brand management activities for our organization
- To understand the current questions, concerns and information voids in different online communities and produce rapid content to respond to them
- To mine for mentions on our organization, topic or campaign To conduct scientific research
- To conduct open sources monitoring for epidemic intelligence
- Other: ______________________________________________

1. **When you produce reports based on analysis of social media do you formulate recommendations for action? Please choose only one of the following:**

- Yes
- No
- I don't know

1. ***(If Yes to 21)* Please tell us what currently happens with the reports you produce.**

|  | **Yes** | **No** | **I Don’t know** |
| --- | --- | --- | --- |
| Is there discussion about these recommendations? |  |  |  |
| Are they sent to internal colleagues for action? |  |  |  |
| Are they sent to external colleagues for action? |  |  |  |
| Do you get feedback on the actions generated? |  |  |  |
| Are they included in any standing meetings, reports or situation reports? |  |  |  |
| Do you track any follow-up actions from the report recommendations? |  |  |  |

1. ***(If no to 21)* Please tell us why you do not produce reports and recommendations. Please choose all that apply:**

- Don’t have time
- Don’t know how to
- It is not required / wanted by report receivers.
- Other: _____________________

**Strengthening social media analysis for public health (Section 4/4)**

These questions ask for your thoughts on how we could strengthen social media analysis for public health.

1. **Please rank in order your top 5 criteria with choosing a social media analysis tool for public health. Top is most preferred. Please choose your top 5.**

- Cheap or free to use
- Familiarity of our team with the tool
- Easiest to procure for our team
- All colleagues have access to same platform
- Broad inclusion of many digital sources
- Ability to easily generate shareable reports
- Ability to integrate off-line sources
- Available in multiple languages
- Can set up a public health taxonomy to filter and export content into
- Ability to export data and annotations
- Transparency of underlying data sourcing, and algorithms for automated analysis and coding
- Includes AI functionality to automate some processes and analyses Filterable by country to prioritise certain regions or countries
- Filters and categorisation for easier insight generation
- Enables adding custom Boolean search strings

1. **What support do you or your team need to better analyze social media for public health action? Please choose all that apply:**

- Training on platform use
- Training on developing search strategies
- Training on sampling, data source selection and analysis plan definition
- Training on integrating social media insights with other data sources (including categorising and organizing data)
- Training on report and recommendation development and writing
- Support from managers
- Access to better platforms
- Access to appropriate IT equipment and internet connection
- Better ability to integrate offline content
- Access to better coverage of locally relevant digital data
- Support in monitoring and evaluation
- Support from regional social listening focal points, or subject matter experts
- Training on how social media analysis fits into wider infodemic management strategies
- More human resources
- Other: __________________________

1. **Is there anything else you would like to tell us about social media monitoring tools or combining the social media insights with other data sources for public health action?**

Please write your answer here: __________________________________
