# Supplementary material for: Informing social media analysis for public health: a cross-sectional survey of professionals
Source: Arch Public Health. 2024 Jan 2;82:1. doi: 10.1186/s13690-023-01230-z (PMC10759433; doi:10.1186/s13690-023-01230-z)
Supplement: Supplementary file 2 — Additional file 2. Full list of responses to open-text question asking final thoughts mapped to themes. [file 13690_2023_1230_MOESM2_ESM.docx]

Additional file 2. Full list of responses to open-text question asking final thoughts mapped to themes.

Please note, we have altered slightly in some instances to remove identifying features. In several cases where the entry was two-part, we have separated into different categories.

| **Theme** | **Description** | **Comment provided** |
| --- | --- | --- |
| Increasing resourcing and collaboration | A need for collaboration, and coordination at global at regional levels. | *To excel in this area, we need to have trained dedicated resources at country offices to work on this area themselves and to work with the national counterparts to strengthen their capacities as well.* |
|  |  | *suggest looking at a new tool developed at XXX - it's freely available and pulls data for thematic analysis from Reddit but can be expanded to Twitter and with hopes, other platforms.* |
|  |  | *Implement XXX in all African countries for regional context.* |
|  |  | *It has been hard to find a way to integrate what the brand needs versus what the technical units need. Technical units need monitoring for other issues and particular searches for data gathering. Currently, there isn't a data intelligence department or team or capabilities for the technical units. Social media is just one piece of the puzzle, we would like to know what is happening in terms of information and how this affects our department and activities and strategize with research backing our decisions.* |
|  |  | *It will be good if we had repository of analysis that has been done previously.* |
|  |  | *Sustain the integration of the routine social listening in routine immunization.* |
|  |  | *the uk has not embraced the who infodemic managers course and i feel we could and should form a network.* |
|  |  | *We find that managing expectations is important. Social media analyses are research tools that can be used to turn data into information. However, there are limits in its applicability, the start up is time (and HR) consuming and its results are sometimes ambigious, similar to other types of research.* |
|  |  | *Yes, XXX has direct links to communities (boots on the ground) through extensive partnerships. XXX should seriously consider how to integrate XXX strategic advantages with XXX social listening objectives.* |
| Recognising value | Several comments related to managerial barriers and the need to build capacity of those managing infodemic teams to recognise the value. | *Managers tend to expect way more results for way less money. Social media analysis is a multidisciplinary venture: data, linguistics, psychology, media-expertise, all is needed to optimally get the potential. This makes it a challenge to acquire and maintain the teams.* |
|  |  | *I am needing support to help demonstrate value for local public health units because the comms teams are resisting.* |
|  |  | *More people should come on board for the work should move well and also there should be some motivation for the worker.* |
|  |  | *Social media monitoring and responding to emerging misinformation or disinformation trends is not seen as a core public health function. It is seen as something to be done by communication team. The communication team sees their role as predominantly reputation managers, working mostly with a handful of mainstream media corporations. The result is that there is no systematic attempt to do anything about what goes on social media.* |
|  |  | *Social media monitoring and responding to emerging misinformation or disinformation trends is not seen as a core public health function. It is seen as something to be done by communication team. The communication team sees their role as predominantly reputation managers, working mostly with a handful of mainstream media corporations. The result is that there is no systematic attempt to do anything about what goes on social media.* |
| Advocacy and strategic leadership | Comments in this theme addressed advocacy and leadership needed to advance infodemic management. This included advocacy with platforms. | *Sometimes we have the management support and the colleagues willingness to do the analysis or provide the data, and also there are existing platforms that can provide most of our requirements, however the legal process is not as fast as we need or teams cannot use or move forward with a platform for legal processes.* |
|  |  | *We need help to PRESSURE these social media platforms, to open up access to RESEARCHERS - so in turn we can better help make things better in their platform.* |
|  |  | *Most managers in surveillance programs or national associations that utilize social media are not going past the "counting clicks" part. They aren't using digital data for analysis and recommendation sharing. I look forward to have this advanced in 2023 in collaboration with local, regional and national orgs. : )* |
|  |  | *National associations have an AMAZING social media presence with comments posted - but not being analyzed or integrated to my knowledge. Universities, I don't know who to talk to yet. Any feedback would be great!* |
|  |  | *Sometimes we have the management support and the colleagues willingness to do the analysis or provide the data, and also there are existing platforms that can provide most of our requirements, however the legal process is not as fast as we need or teams can not use or move forward with a platform for legal processes. Is there a way to review the related policies? Thank you* |
|  |  | *Managers tend to expect way more results for way less money. Social media analysis is a multidisciplinairy venture: data, linguistics, psychology, media-expertise, all is needed to optimally get the potential. This makes it a challenge to acquire and maintain the team.* |
| Digital and technical barriers | Some respondents discussed wanting better access to data and to better platforms. Others discussed challenges with internet and VPN access, as well as a lack of data for certain priority groups. | *It's still very hard to obtain USABLE data from various social media platforms - TikTok, Facebook, Instagram. For example, we tried to create a crawler for Instagram, to be able to better monitor the infodemic situation in that platform. Instead, we were hit by various very harsh anti-bot measures. We should not need to create the crawler at the first place, but since these platforms are so bent on closing off their places, we had to.* |
|  |  | *Internet connection is the most important and data.* |
|  |  | *Challenges in countries that have banned some social media platforms, hence when the citizens use VPN, to access such platforms country of origin is altered.* |
|  |  | *Adapting social media monitoring to fully capture the underserved riverine communities in Nigeria.* |
|  |  | *It's very difficult to get data based on rase or ethnicity. My agency targets the Black American audience, which has lower vaccination rates that other communities. We can't filter users by race/ethnicity, and there are very few community reports about vaccination rates that do this. So, a lot of it involves making guesses about race, or using other identifiers to assume their race. I wish there was a way to get more data based on race.* |
|  |  | *Social media coverage is low in Afghanistan, due lack of access to internet.* |
|  |  | *The social media monitoring tool is unfortunately misused and that increases mistrust of publics toward to them.* |
|  |  | *Access to premium SM monitoring tools would benefit immensely.* |
|  |  | *Public Health is the backbone of Health Delivery System, so therefore means of data sourcing and its capacity building resources must be made available and cheap to enable creating of wealth, since Health is wealth.* |
|  |  | *There should be a free access accounts for researchers, data from countries with conflict settings are sometimes restricted and we need access to them since we are working for the sake of research and science.* |
|  |  | *We use our own systems that we have developed on our own, not third party tools, so some answers are not relevant to our situation.* |
| Teaching and evidence | These comments included support for those teaching university level students, as well as calls for computer literacy and social inoculation. | *I would like to know any relevant publications which use social media monitoring tools, and how to develop materials for teaching social media monitoring in basic level to equip health cadres with ability to track mis/disinformation.* |
|  |  | *I teach MPH public health policy, and the importance of the use of good data to justify the policy action. So I am primarily instructing my students on access to use these resources. This educational route was not really mentioned in your survey.* |
|  |  | *We find that managing expectations is important. Social media analyses are research tools that can be used to turn data into information. However, there are limits in its applicability, the start up is time (and HR) consuming and its results are sometimes ambigious, similar to other types of research.* |
|  |  | *We summarized our strategy/plans in this preprint <link>.* |
|  |  | *Social media is the key for social inoculation of the communities for wealth creation, since Health is wealth.* |
|  |  | *The current infodemiological situation denotes the stringent urgency to create infodemic resilience in people (we cannot properly counter dis-misinformation by only generating new information). To the best of my scientific knowledge, it is essential to start school educational programs (from kindergarten to university), adopting preparatory games and exercises to train the critical scientific sense (rational sphere) and ability to manage information overload (emotional sphere). I am trying to tackle this project, but I need support from health authorities like yours. In this regard, I believe that monitoring can be useful for formulating an educational project that is contextualized in the current infodemiological scenario.* |
|  |  | *Computer literacy is a necessity even for professionals with postgraduate degrees in my country, but they lack interest, considering it not necessary, and they replicate bad practices.* |
| Technical training needs | Respondents detailed technical training needs for themselves and the sector, and called for training to be offered in a range of modalities. | *Better to work on the following: These are the key social media metrics that should not miss from your social media analysis report: post ranks across all channels. number of posts. post engagement and engagement rate. all campaigns. total campaign engagement. number of posts per campaign. posts volumes by campaign (numerically and percentage wise).* |
|  |  | *I would like more training through MOOC.* |
|  |  | *I am not precisely working on "Social Media" but rather on circulation of international news flow on the internet. I strongly prefer to use RSS flows of daily newspaper rather than Twitter or Facebook data ...* |
|  |  | *I need to have more familiarity on these software for social media analysis.* |
|  |  | *I wish if I had an easy access to platforms such as Facebook during conducting my thesis project that would help me in monitoring people’s insights on the COVID-19 vaccine and Infodemic. I wish if there is a training for better managing social media data.* |
|  |  | *A certificate should be provided after the training/course.* |
|  |  | *I wish if I had an easy access to platforms such as Facebook during conducting my thesis project that would help me in monitoring people’s insights on the COVID-19 vaccine and Infodemic. I wish if there is a training for better managing social media data.* |
|  |  | *Tt has been hard to find a way to integrate what the brand needs versus what the technical units need. Technical units need monitoring for other issues and particular searches for data gathering. Currently, there isn't a data intelligence department or team or capabilities for the technical units. Social media is just one piece of the puzzle, we would like to know what is happening in terms of information and how this affects our department and activities and strategize with research backing our decisions.* |
|  |  | *Require training of public health staff to use social media for our activities.* |
|  |  | *Most managers in surveillance programs or national associations that utilize social media are not going past the "counting clicks" part. They aren't using digital data for analysis and recommendation sharing. I look forward to have this advanced in 2023 in collaboration with local, regional and national orgs. : )* |
|  |  | *Training will be useful Convenient platform for social media monitoring.* |
